# Supplementary material for: The Sum of Plasma Fatty Acids iso16:0, iso17:0, trans11-18:1, cis9, trans11-CLA, and cis6-18:1 as Biomarker of Dairy Intake Established in an Intervention Study and Validated in the EPIC Cohort of Gipuzkoa
Source: Nutrients. 2021 Feb 22;13(2):702. doi: 10.3390/nu13020702 (PMC7926849; doi:10.3390/nu13020702)
Supplement: Supplementary file 1 [file nutrients-13-00702-s001.pdf]

Supplementary Material

Table S1. Participant's baseline anthropometric and diet characteristics in the intervention and the observational studies, mean (SD).

Intervention Study		Observational Study				
		Dairy Consumption, g/day				
	382.2 (123.2) [1]	<0.71	9.37–217.74	221.43–351.25	>351.25	*p*-value [2]
n	10	35	38	38	40	
Sex						
Male	2	28	13	12	15	
Female	8	7	25	26	25	
Age at recruitment, year	42.8 (11.7)	49.5 (7.5)	45.4 (7.2)	45.9 (7.4)	47.9 (7.2)	0.067
BMI, kg/m^2	22.6 (2.8)	28.7 (2.7)	26.9 (4.3)	26.8 (3.3)	27.1 (4.2)	0.104
Total energy, Kcal	1620.7 (247.4)	2442.9 (726.1)	2092.8 (773.6)	2103.9 (586.7)	2343.2 (582.2)	0.060
Food, g/day						
Fruits, nuts and seeds	419.2 (151.6)	416.0 (346.0)	305.7 (259.6)	368.6 (285.0)	355.0 (200.1)	0.398
Vegetables	205.95 (75.8)	254.47 (25.3)	239.65 (21.7)	229.17 (22.9)	232.73 (22.3)	0.798
Meat and meat products	97.6 (50.7)	185.0 (82.5) [a]	156.0 (100.6) [a,b]	130.7 (64.6) [b]	141.2 (64.8) [a,b]	0.025
Red meat	34.8 (21.8)	87.8 (59.1) [a]	74.1 (71.8) [a,b]	48.2 (32.3) [b]	47.9 (32.2) [b]	0.001
Egg and egg products	22.2 (9.71)	35.9 (29.6)	31.7 (27.5)	24.6 (15.2)	34.9 (15.2)	0.128
Fish and seafood	84.3 (29.7)	86.59 (62.5) [a]	76.94 (49.9) [a,b]	58.90 (36.8) [b]	58.93 (34.0) [b]	0.018
Fat[3]	25.2 (5.4)	36.3 (12.1)	32.2 (14.6)	31.3 (13.0)	33.0 (13.3)	0.415
Sugar and confectionary	26.9 (20.9)	14.5 (17.9)	15.9 (12.7)	23.9 (21.5)	23.5 (22.8)	0.065
Cakes and biscuits	32.6 (21.1)	11.1 (16.9) [a]	23.1 (30.5) [a,b]	21.8 (27.9) [a,b]	32.8 (43.8) [b]	0.035
Olive oil	25.2 (5.45)	17.5 (18.7)	18.8 (13.2)	14.2 (1.6)	16.7 (16.5)	0.619
Main nutrients [4], energy%						
Proteins	78.3 (14.8), 17.6%	117.2 (32.6), 19.5%	103.0 (38.1), 20.0%	101.0 (28.8), 19.5%	114.2 (28.0), 19.8%	0.078
Lipids	72.2 (13.2), 36.6%	81.2 (27.7), 30.3%	82.5 (27.9), 36.4%	83.4 (29.8), 35.5%	91.7 (31.7), 34.8%	0.386
Carbohydrates	152.9 (29.2), 40.9%	246.5 (84.9) [a], 40.7%	201.7 (74.2) [b],38.5%	212.1 (62.4) [a,b],40.9%	249.1 (62.8) [a], 42.9%	0.006
Alcohol	12.4 (13.9), 4.9%	36.7 (33.4) [a], 9.6%	18.8 (27.9) [a,b], 5.1%	14.1 (19.1) [b], 4.1%	9.3 (16.1) [b], 2.5%	<0.001

[1] In the habitual diet of participants. [2]*p*-values corresponding to the observational study. [3] Olive oil and other oils and fats using for cooking. [4] Calculated from the content of ingested food using food composition tables [11]. [a, b] different letters indicate significant differences ($p \leq 0.05$) in compared groups within the observational study.

Table S2. Fatty Acids concentration, µmol/L, mean (SD) in erythrocytes in participants before (Baseline) and after the intervention study with a diet without dairy products (No Dairy) and a diet with dairy products (With Dairy).

Fatty Acids	Baseline	No Dairy	With Dairy	*p* [1]
MCSFA				
10:0	30.95 (2.96)	31.18 (3.05)	31.75 (1.82)	0.599
12:0	6.760 (3.33) [a]	11.89 (7.21) [a,b]	11.39 (1.61) [b]	0.012
14:0	17.38 (5.15) [a]	13.12 (3.88) [b]	26.14 (7.56) [c]	<0.001
15:0	92.77 (10.87)	83.59 (19.06)	98.92 (25.96)	0.102
Sum	147.9 (15.23) [a]	139.8 (25.89) [a]	168.2 (28.14) [b]	0.005
LCSFA				
16:0	798.6 (73.01) [a]	733.5 (114.62) [a]	900.1 (126.34) [b]	<0.001
17:0	9.840 (1.54) [a]	8.483 (1.87) [b]	11.49 (2.55) [c]	<0.001
18:0	583.8 (51.29)	546.3 (92.08)	622.9 (95.78)	0.050
20:0	4.715 (1.31)	4.251 (0.87)	4.599 (0.74)	0.372
21:0	1.560 (0.28)	1.701 (0.45)	1.736 (0.29)	0.329
22:0	2.603 (0.57) [a]	2.607 (0.51) [a]	3.442 (1.23) [b]	0.006
24:0	2.959 (0.83)	2.431 (0.50)	2.617 (0.76)	0.044
26:0	41.68 (5.57)	37.53 (7.93)	45.16 (13.01)	0.039
Sum	1446 (111.53) [a]	1337 (206.80) [a]	1592 (210.03) [b]	0.003
ΣSFA	1594 (121.65) [a]	1477 (223.14) [a]	1760 (227.03) [b]	0.003
ΣOCFA	104.2 (11.29)	93.78 (20.29)	112.1 (26.73)	0.064
BCSFA				
iso14:0	5.360 (1.58)	4.628 (1.56)	4.402 (2.45)	0.098
anteiso14:0	2.543 (0.81)	2.749 (1.01)	3.228 (0.84)	0.086
iso15:0	62.30 (17.68)	54.95 (19.14)	50.81 (27.21)	0.025
anteiso15:0	0.7857 (0.23)	0.7281 (0.32)	0.9002 (0.21)	0.099
iso16:0	17.07 (4.21)	14.25 (4.51)	13.64 (7.01)	0.022
iso17:0	5.268 (1.91) [a]	4.018 (1.26) [b]	7.506 (2.90) [c]	<0.001
anteiso17:0	4.091 (0.63)	4.623 (1.09)	5.070 (2.16)	0.051
Sum	97.42 (21.86)	86.11 (25.29)	85.69 (34.88)	0.071
c-MUFA				
cis9-14:1	6.644 (2.23) [a]	5.090 (1.11) [b]	8.875 (2.53) [c]	<0.001
cis9-16:1	17.62 (8.44) [a,b]	15.41 (6.18) [a]	20.92 (8.63) [b]	0.007
cis10-17:1	24.33 (5.75)	22.73 (7.88)	26.18 (8.98)	0.279
cis6-18:1	2.583 (0.61) [a]	1.564 (0.30) [b]	3.115 (0.83) [c]	<0.001
cis9-18:1	545.4 (69.70) [a]	544.7 (138.05) [a]	642.2 (154.02) [b]	<0.001
cis11-18:1	51.47 (5.79) [a]	54.74 (12.81) [a,b]	59.46 (11.31) [b]	0.021
cis11-20:1	7.120 (0.89)	7.367 (2.20)	7.955 (1.51)	0.113
cis13-22:1	66.16 (11.69) [a]	54.75 (7.73) [b]	75.77 (20.69) [c]	<0.001
Sum	724.8 (84.17) [a]	709.6 (163.80) [a,b]	848.5 (175.67) [b]	<0.001
t-MUFA				
trans10-15:1	1.421 (0.37) [a,b]	1.235 (0.29) [a]	1.535 (0.47) [b]	0.024
trans9-16:1	1.627 (0.53)	1.627 (0.72)	2.101 (1.31)	0.113
trans9-18:1	2.030 (0.40) [a]	1.943 (0.42) [a]	2.457 (0.43) [b]	<0.001
trans11-18:1	2.454 (0.77) [a]	1.641 (0.49) [b]	3.025 (0.95) [c]	<0.001
Sum	7.531 (1.36) [a]	6.446 (1.07) [b]	9.118 (2.21) [c]	<0.001

PUFA				
*cis*9,*cis*12-18:2	606.3 (122.05) [a]	583.7 (100.85) [a]	743.5 (251.43) [b]	0.001
18:3 ω-6	9.932 (2.47) [a,b]	8.033 (2.87) [a]	12.93 (9.69) [b]	0.032
18:3 ω-3	4.692 (1.98) [a,b]	3.808 (1.32) [a]	6.396 (3.27) [b]	<0.001
20:4 ω-6	1092 (192.44)	1011 (244.44)	1174 (175.81)	0.029
20:5 ω-3	43.87 (22.58) [a]	43.96 (31.10) [a]	60.13 (30.94) [a]	<0.001
22:6 ω-3	399.5 (53.76)	384.2 (99.14)	442.6 (82.38)	0.060
Sum	2157 (250.87) [a]	2035 (347.43) [a]	2440 (370.50) [b]	0.002
CLA				
cis9,trans11-CLA	4.097 (1.17) [a]	2.562 (0.79) [b]	5.498 (1.84) [c]	<0.001
trans10,cis12-CLA	9.775 (3.40) [a]	9.456 (3.05) [a]	12.72 (1.64) [b]	0.001
cis9,cis11-CLA	3.612 (1.44)	3.059 (0.76)	3.473 (0.78)	0.216
trans9,trans11-CLA	1.219 (0.22) [a,b]	1.083 (0.20) [a]	1.387 (0.28) [b]	<0.001
Sum	18.70 (3.07) [a]	16.16 (3.06) [b]	23.08 (3.62) [c]	<0.001
TOTAL	4595 (414.57) [a]	4327 (710.22) [a]	5163 (700.85) [b]	0.001

[1] *p*-value < 0.05 was considered to indicate statistical significance. [a, b, c] different letters indicate significant differences in compared groups. MCFA, medium chain saturated fatty acids; LCSFA, long chain saturated fatty acids. ΣSFA, sum of saturated fatty acids; ΣOCSFA, sum of odd numbered chain saturated fatty acids; BCFA, branched-chain fatty acids; c-MUFA, *cis*-monounsaturated fatty acids; t-MUFA, *trans*-monounsaturated fatty acids; PUFA, polyunsaturated fatty acids; CLA, conjugated linoleic acids.

Table S3. Ratio between plasma fatty acids concentration and ingested red meat in the observational and intervention studies.

	FA/red meat[1]	
**Fatty acid**	**Intervention Study**	**Observational Study**
iso16:0	0.124 ± 0.051	0.098 ± 0.054
iso17:0	0.221 ± 0.119	0.250 ± 0.134
trans11-18:1	0.108 ± 0.084	0.101 ± 0.072
cis6-18:1	0.025 ± 0.012	0.033 ± 0.034
cis9,trans11-CLA	0.163 ± 0.115	0.237 ± 0.149
Sum[2]	0.640 ± 0.360	0.718 ± 0.408

[1] Corresponding fatty acid concentration (μmol/L) in plasma of samples from participants who did not consume dairy products divided by ingested red meat (g/day), mean ± SD. [2] ∑iso16:0; iso17:0; 17:0; *trans11*-18:1; *cis9,trans11*-CLA; *cis6*-18:1. CLA, conjugated linoleic acid.

Table S4. Biochemical serum parameters for the samples taken in the intervention study and the observational study.

Biochemical Parameter	Intervention Study				Observational Study				
	Baseline	No Dairy	With Dairy	p [1]	Q0	Q1	Q2	Q3	p [1]
TG, mg/dL	77.3 (35.6)	81.0 (33.5)	82.4 (35.0)	0.739	75.8 (32.9)	68.7 (25.5)	65.1 (21.3)	66.4 (32.6)	0.393
TC, mg/dL	246.3 (39.5)	237.5 (37.8)	265.1 (46.3)	0.079	161.8 (31.5)	164.8 (24.5)	163.8 (24.9)	160.8 (30.2)	0.917
LDL-C, mg/dL	117.5 (24.2)	105.9 (28.6)	115.2 (28.9)	0.491	98.9 (26.3)	100 (21.9)	99.4 (18.3)	99.4 (24.5)	0.994
HDL-C, mg/dL	88.0 (19.9)	87.7 (19.7)	87.4 (23.9)	0.983	47.8 (10.8)	50.7 (9.4)	51.4 (11.1)	48.1 (10.1)	0.319
TC/HDL-C	2.85 (0.44)	2.78 (0.58)	3.16 (0.76)	0.702	3.45 (0.77)	3.34 (0.69)	3.26 (0.58)	3.45 (0.81)	0.556
AIP	−0.71 (0.25)	−0.70 (0.22)	−0.66 (0.22)	0.243	−0.18 (0.18)	−0.25 (0.20)	−0.27 (0.17)	−0.25 (0.23)	0.288

[1] p-value < 0.05 was considered to indicate statistical significance. TG, triglycerides, TC, total cholesterol, LCL-C cholesterol in low-density lipoproteins, HDL-C, cholesterol in high-density lipoproteins, AIP, Atherogenic Index of Plasma, log (TG/HDL-C).
